# P-303. Linkage to Comprehensive HIV Prevention Services at Prisma Community Care

**DOI:** 10.1093/ofid/ofaf695.523

**Published:** 2026-01-11

**Authors:** Heather Alejandro, Michael Greathouse, Chase Redington

**Affiliations:** Prisma Community Care, Phoenix, AZ; Prisma Community Care, Phoenix, AZ; Prisma Community Care, Phoenix, AZ

## Abstract

**Background:**

Over the past decade, Maricopa County has experienced a rise in new HIV and sexually transmitted infection (STI) diagnoses. Prisma Community Care has intensified its commitment to promoting sexual health and health equity, with an emphasis on removing barriers to care. The Clinical Programs Department at Prisma plays a pivotal role in the HIV Care Continuum by improving linkage to care and expanding access to comprehensive preventative services (CPS) through its Prevention and Navigation programs.

**Figure:**
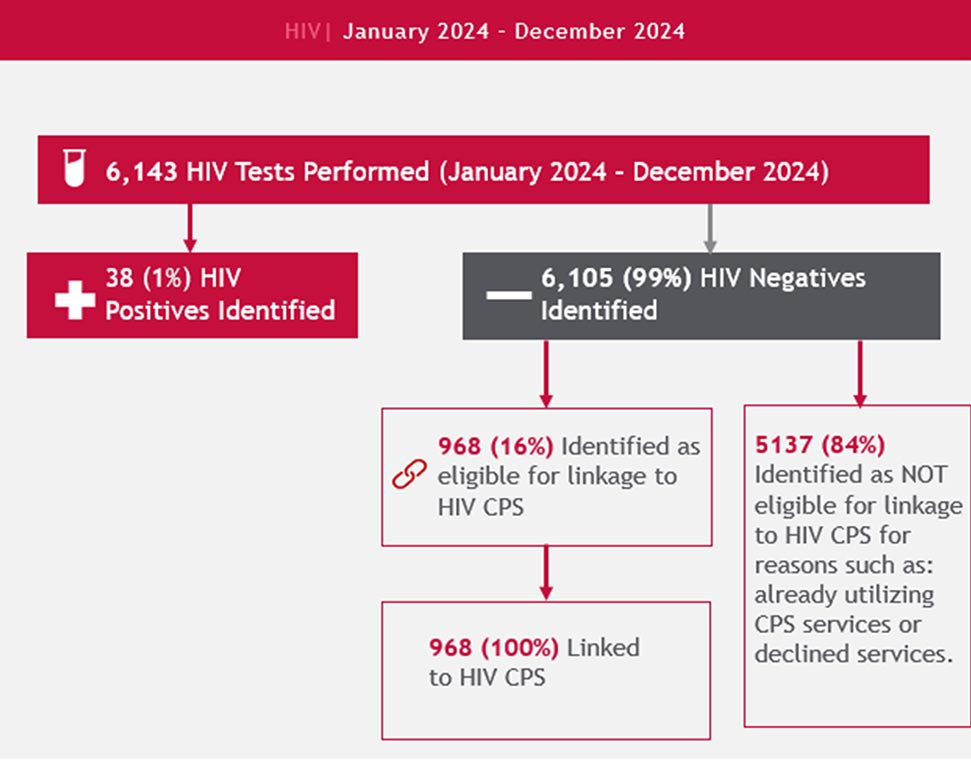
HIV CPS and Linkage to Care

**Figure:**
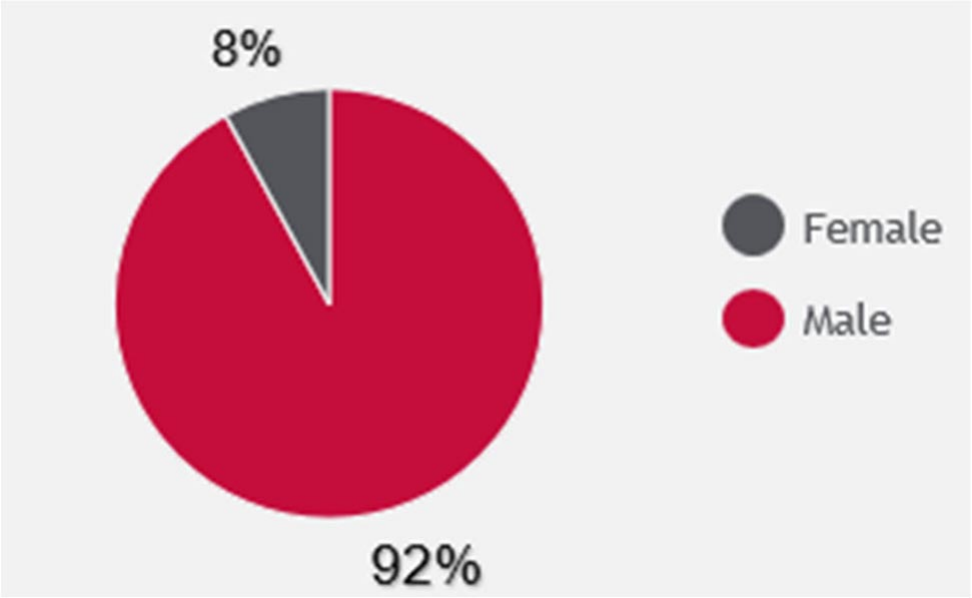
Demographic Data for those Eligible for Linkage to HIV CPS - Gender Out of the 968 patients linked to HIV CPS assessment, 92% identified as male.

**Methods:**

The Prevention and Navigation programs utilize a status-neutral approach, ensuring holistic support that adapts to the specific needs and goals of each client. During a no-cost clinic visit, Prevention Specialists conduct interviews and assessments, offering resources to address barriers. Patients with barriers are referred to Clinical Program Navigators, who furnish patients with knowledge and resources. If a patient tests preliminarily positive for HIV, immediate connection to a Navigator facilitates expedited care, with linkage completed within five days of the result.

**Figure:**
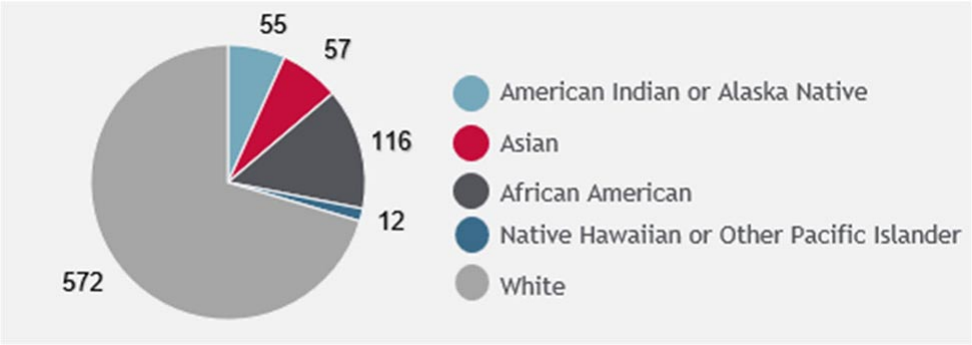
Demographic Data for those Eligible for Linkage to HIV CPS - Race and Ethnicity Most patients linked were White with 58% identifying as Non-Hispanic.

**Figure:**
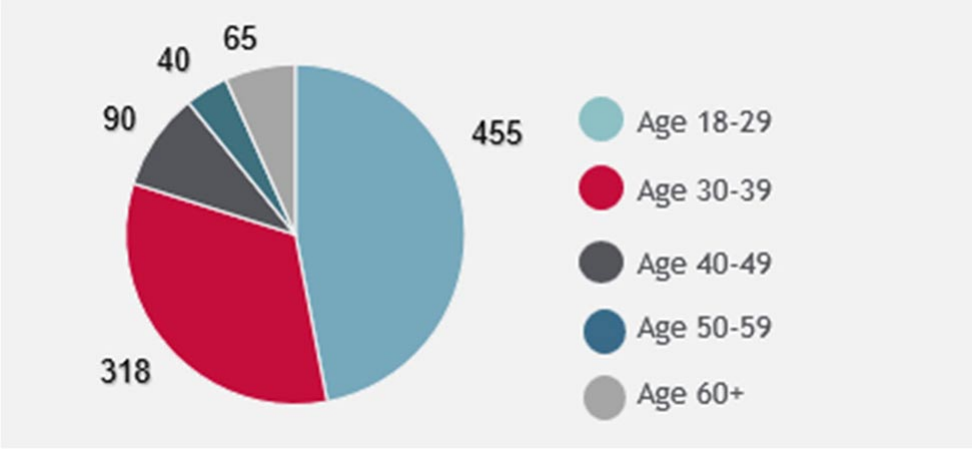
Demographic Data for those Eligible for Linkage to HIV CPS - Age A majority of linked patients were between 18 and 29 years of age.

**Results:**

Between January 2024 - December 2024, Prisma has tested 6,143 individuals for HIV. Among the tests, 6,105 patients received negative results. Assessments and evaluations were conducted for 968 of those negative clients and found eligible for CPS. Out of the 968 patients deemed eligible, 100% of them were linked to CPS and attended their first visit.

**Conclusion:**

By focusing on key elements of the HIV Care Continuum, such as linkage and retention, there are new opportunities to define roles and tasks that enhance the quality of service delivery. Providing time and space to build rapport with patients fosters their openness to education about HIV CPS services. Additionally, improving staff education around sensitivity and status-neutral approaches positively influences patients’ long-term engagement with Prisma staff.

**Disclosures:**

All Authors: No reported disclosures

